# *SERPINE2 *haplotype as a risk factor for panlobular type of emphysema

**DOI:** 10.1186/1471-2350-12-157

**Published:** 2011-12-07

**Authors:** Mari K Kukkonen, Emmi Tiili, Satu Hämäläinen, Tapio Vehmas, Panu Oksa, Päivi Piirilä, Ari Hirvonen

**Affiliations:** 1 Finnish Institute of Occupational Health, Helsinki, Finland; 2 Helsinki University Hospital, Department of Clinical Physiology, Helsinki, Finland

## Abstract

**Background:**

*SERPINE2 *(serpin peptidase inhibitor, clade E, member 2) has previously been identified as a positional candidate gene for chronic obstructive pulmonary disease (COPD) and has subsequently been associated to COPD and emphysema in several populations. We aimed to further examine the role of *SERPINE2 *polymorphisms in the development of pulmonary emphysema and different emphysema subtypes.

**Methods:**

Four single nucleotide polymorphisms (SNPs) in *SERPINE2 *were analyzed from 951 clinically and radiologically examined Finnish construction workers. The genotype and haplotype data was compared to different emphysematous signs confirmed with high-resolution computed tomography (HRCT), forced vital capacity (FVC), forced expiratory volume in one second (FEV_1_), diffusing capacity (DL_CO_), and specific diffusing capacity (DL_CO_/VA).

**Results:**

Three of the studied *SERPINE2 *SNPs (rs729631, rs975278, and rs6748795) were found to be in tight linkage disequilibrium. Therefore, only one of these SNPs (rs729631) was included in the subsequent analyses, in addition to the rs840088 SNP which was in moderate linkage with the other three studied SNPs. The rs729631 SNP showed a significant association with panlobular emphysema (*p *= 0.003). In further analysis, the variant allele of the rs729631 SNP was found to pose over two-fold risk (OR 2.22, 95% CI 1.05-4.72) for overall panlobular changes and over four-fold risk (OR 4.37, 95% CI 1.61-11.86) for pathological panlobular changes. A haplotype consisting of variant alleles of both rs729631 and rs840088 SNPs was found to pose an almost four-fold risk for overall panlobular (OR 3.72, 95% CI 1.56-8.90) and subnormal (OR 3.98, 95% CI 1.55-10.20) emphysema.

**Conclusions:**

Our results support the previously found association between *SERPINE2 *polymorphisms and pulmonary emphysema. As a novel finding, our study suggests that the *SERPINE2 *gene may in particular be involved in the development of panlobular changes, *i.e.*, the same type of changes that are involved in alpha-1-antitrypsin (AAT) -deficiency.

## Background

Pulmonary emphysema is a smoking associated condition of the lung, which often develops as a component of chronic obstructive pulmonary disease (COPD). In addition to emphysema, COPD encompasses chronic bronchitis and small airway disease [1]. Cigarette smoking is the main environmental risk factor for COPD, but the disease is also likely influenced by several genes and gene-smoking interactions.

To date, the only proven genetic risk factor for COPD is the severe deficiency of alpha-1-antitrypsin (AAT), which predisposes to early onset panacinar (panlobular) type of emphysema [2,3]. AAT, a serine protease inhibitor encoded by the *SERPINA1 *gene, has a major role in inactivating neutrophil elastase and other proteases thereby maintaining the protease-antiprotease balance. Disturbance of this balance is believed to explain lung destruction in emphysema [4,5].

Another member of the serpin-family, *SERPINE2 *(serpin peptidase inhibitor, clade E [nexin, plasminogen activator inhibitor type 1] member 2) was identified as a COPD candidate gene using gene expression analysis of murine and human lung tissues [6]. In the same study, several single nucleotide polymorphisms (SNPs) in *SERPINE2 *gene were associated to COPD in family and case-control-based study populations. Subsequently, the association between the *SERPINE2 *SNPs and COPD has been replicated in two large studies in Caucasian populations (family and case-control-based) [7] and in one study in a Korean population [8]. On the other hand, the association has also been failed to be replicated in one Caucasian [9] and one Chinese [10] study.

Since COPD is a heterogenic disease with the inclusion criteria varying greatly between different studies, the exact phenotype, to which *SERPINE2 *gene contributes to, has remained ambiguous. Recently, however, one research group has shed light on this issue by examining the *SERPINE2 *polymorphisms in relation to computed tomography (CT) quantified emphysema and airway wall phenotypes [11]; interestingly, associations were found between several *SERPINE2 *polymorphisms and densitometric emphysema. In addition, another research group recently associated *SERPINE2 *polymorphism with autopsy diagnosed emphysema among Japanese smokers [12].

We aimed to verify the potential association between the *SERPINE2 *gene variations and radiologically defined emphysema, and to study further the potential role of *SERPINE2 *polymorphisms in the development of airway limitation and different emphysema subtypes among Finnish Caucasian construction workers. Signs of different emphysema types (centrilobular, paraceptal, panlobular, and bullae) were determined from all the study subjects by using the high-resolution computed tomography (HRCT). In addition, lung function was examined using spirometry and diffusing capacity measurements. Based on previous association studies, four SNPs in the *SERPINE2 *gene (rs729631, rs975278, rs6748795, and rs840088) were chosen to be studied in relation to the above factors.

## Methods

### Study population

This study combines the study populations from two previous screening studies aiming to detect early occupational chest diseases among asbestos exposed workers. The first study group (ASBE, n = 602) was recruited in 1996-1997 and consisted of asbestos exposed subjects who lived in Helsinki area, were willing to participate, and had asbestosis with or without smoking, or bilateral pleural plaques without asbestosis and a smoking history for at least 10 years [13,14]. The second study group (ASSE, n = 633) was recruited in 2003-2004 and consisted of asbestos exposed subjects from three geographic areas (Helsinki, Tampere, and Turku), who had participated in the asbestos screening program in 1990-1992 and were heavily exposed, or who had previously been diagnosed with asbestos related occupational disease and had visited wards of occupational medicine in Helsinki and Tampere for a clinical follow-up [15].

Altogether 178 of the subjects recruited in 2003-2004 had already participated in the first examination in 1996-1997. They were therefore excluded from the second patient group in the present study before combining the data. In the combined study population, blood samples were available from 1021 subjects, 1013 of whom the genotyping data was successfully achieved. However, 25 more subjects were excluded because of missing smoking information and 37 because of insufficient asbestos exposure data. Thus, the final study group consisted of 951 subjects (935 males, 16 females).

An approval for the study was obtained from the local ethics committee according to the legislation at the time of the original study. All subjects gave an informed consent to participate in the study.

### Radiological examinations

The lungs of the construction workers were imaged prone in full inspiration with four different scanners: in 1996-1997 the Picker PO 2000 (Picker International, Cleveland, USA) device was used, whereas in 2003-2004 Siemens Somatom Balance (Siemens Medical, Erlangen, Germany) was used in Helsinki, Siemens Somatom Plus 4 (Siemens Medical) was used in Tampere, and GE Light-speed 16 Advantage (GE Healthcare, Milwaukee, WI, USA) was used in Turku.

The HRCT images were printed as hard copies and analyzed blindly by two (2003-2004) or three (1996-1997) radiologists. Emphysema was defined as a sharply delineated low-density area according to the criteria and reference images of Webb *et al. *[16]. Signs of centrilobular, paraceptal, panlobular, and bullae-type emphysema was scored in both lungs by using a scale from 0 to 5: 0 (no changes), 1 (faint or subnormal abnormalities, in a single slice or few slices), 2 (slight abnormalities in some slices), 3 (clear abnormalities in several slices), 4 (score between 3 and 5), and 5 (abnormalities widely distributed in the whole lung, in all or most slices). These emphysema subtype scores were added up to form emphysema sum score, the maximum being 20 [14]. Mean scores of both lungs were used in the analysis. The intra- and inter-reader consistencies of readings have previously been reported [13].

### Lung function examinations

Flow-volume spirometry was performed with a rolling-seal spirometer (Mijnhard BV, Bunnik, Holland) connected to a microcomputer (Medikro MR-3; Medikro, Kuopio, Finland), using the reference values of Viljanen [17] and the standards of the European Respiratory Society (ERS) [18]. The following parameters were measured: forced vital capacity (FVC), forced expiratory volume in 1 second (FEV_1_), and the FEV_1_/FVC ratio.

The single breath diffusing capacity for carbon monoxide (DL_CO_) and specific diffusing capacity (diffusing capacity related to alveolar volume DL_CO_/VA) were measured by using a Masterlab Transfer or a Compact Lab Transfer device (Erich Jaeger, Würzburg, Germany) according to ERS recommendations [19]. Correction of DL_CO _was done according to patient's actual hemoglobin level.

The lung function variables, excluding FEV_1_/FVC ratio, were handled as percent of Viljanen reference values [17] based on the distribution of values in the reference population. The DL_CO _and DL_CO_/VA values were considered decreased if they were < 74% of predicted, the FEV_1 _and FVC values were considered decreased if they were < 80% of predicted, and the FEV_1_/FVC ratio was considered decreased if it was < 88% of predicted [17].

### Genotyping analyses

DNA was extracted mechanically using Thermo King Fisher mL (Thermo Fisher Scientific, Erembodegem, Belgium) from whole blood using Biosprint 15 DNA Blood Kit (Qiagen, Hilden, Germany) and stored at -20°C until use.

Three *SERPINE2 *SNPs (rs729631, rs975278, and rs6748795) were genotyped using the OpenArray-system (BioTrove Inc., Woburn, MA, USA), a next-generation quantitative PCR platform based on TaqMan chemistry. The assay IDs for TaqMan^® ^SNP Genotyping Assays spotted on the array were C__803914_10, C__7614671_10, and C__1677432_10, respectively. Plate format of 16 SNPs and 144 samples per array was used. The allele calling analysis was performed using OpenArray™SNP Genotyping Analysis software (BioTrove Inc., Woburn, MA, USA).

For the fourth analysed *SERPINE2 *SNP, rs840088, a TaqMan^® ^SNP Genotyping Assay was purchased from Applied Biosystems (assay ID: C__7614655_10). The genotyping was performed with the Allelic discrimination assay of the Applied Biosystems 7500 Real-Time PCR system (Applied Biosystems, Foster City, CA, USA) according to manufacturer's recommendations. Sequence Detection Software 1.4 was used for the allele calling analysis.

For quality control, two independent readers interpreted the results and a random selection of 10% of all samples was re-tested. No discrepancies were discovered in the replicate tests for rs729631 or rs840088. The error rate was 1% for rs975278 and 2% for rs6748795. In addition, to verify the reliability of OpenArray platform, a random selection of 15% of samples were re-analyzed for rs6748795 SNP with 7500 Real-Time PCR system using TaqMan^® ^SNP Genotyping Assay (Assay ID C__7614655_10). No discrepancies were discovered in the re-analysis.

### Statistical analysis

The associations between genotypes/haplotypes, emphysema, and lung function parameters (FEV_1_, FVC, FEV_1_/FVC, DL_CO_, and DL_CO_/VA) were evaluated by using linear regression analysis. Logistic regression analysis was used to evaluate the potential confounders and to further study the risk for emphysematous changes and their severity with certain genotype. Covariates used in the analysis were: sex, age, pack years (PYs) of smoking, and years of asbestos exposure for emphysema; sex, age, PYs, years of asbestos exposure, and height for FEV_1_/FVC; and PYs and years of asbestos exposure for FEV_1_, FVC, DL_CO_, and DL_CO_/VA. Occasionally lacking height data was replaced by the group mean value (68 replacements).

For stratified analyses on smoking habits the subjects who had smoked less than 25 PYs were categorised to mild smokers, and subjects who had smoked for at least 25 PYs were considered moderate/heavy smokers.

All of the data analyses were performed by using the SPSS version 18.0 (SPSS Inc., Chicago, IL).

The linkage disequilibrium (LD) structure among the four studied *SERPINE2 *SNPs was examined using HaploView program, version 4.2 [20]. The *SERPINE2 *haplotypes were statistically reconstructed from population genotype data by using the PHASE program (version 2.1) with the Markov chain method for haplotype assignments [21].

Our study (n = 951) had 80% power to detect OR from 1.66 to 1.72 depending on the minor allele frequency (21-28%). After stratifying the subjects according to radiologic signs, the OR detected with 80% power ranged from 1.82 to 1.90 (subnormal changes, n = 881) and from 2.17 to 2.26 (pathological changes, n = 832). The calculations were performed by using standard methods and are based on a two-sided alpha of 0.05.

The χ^2 ^analysis was used to test for a deviation from the Hardy-Weinberg equilibrium (HWE).

## Results

The demographics, pulmonary function data and HRCT characteristics of the construction workers are summarized in Table 1. All the studied polymorphisms were in HWE.

**Table 1:** Selected characteristics of the study population

	Mean (SD) or N (%)	Range
Age, years	63.2 (7.3)	38.4-87.0
Male sex	935 (98.3)	
Smoking history		
Never smoker	135 (14.2)	
Ex-smoker	595 (62.6)	
Current smoker	221 (23.3)	
Pack years	20.4 (16.7)	
Years of asbestos exposure	23.9 (10.7)	0.0-129.0
Emphysema score (n = 352^#^)	2.00 (2.4)*	0.13-15.0
Centrilobular (n = 228^#^)	1.23 (1.01)*	0.17-4.0
Paraceptal (n = 170^#^)	1.00 (0.91)*	0.17-4.7
Panlobular (n = 168^#^)	0.93 (0.87)*	0.13-4.3
Bullae (n = 125^#^)	0.70 (0.77)*	0.17-4.17
Lung function, % of predicted		
FEV_1 _(n = 922)	83.4 (18.6)	19.0-138.0
FVC (n = 917)	89.0 (15.9)	33.0-149.0
FEV_1_/FVC in percentage (n = 915)	93.5 (12.9)	28.3-166.7
DL_CO _(n = 912)	91.0 (20.2)	22.0-197.0
DL_CO/_VA (n = 914)	98.3 (18.4)	10.0-157.0

DL_CO _= Single breath diffusing capacity for carbon monoxide; DL_CO/_VA = specific diffusing capacity; FEV_1 _= Forced expiratory volume in 1 second; FVC = forced vital capacity; SD = standard deviation

N = 951 except as noted

^#^Number of subjects with emphysema score positive

*The average score of subjects with positive emphysema score

**Figure 1:**
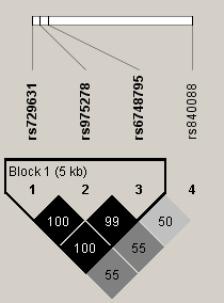
**Linkage disequilibrium (LD) between the studied *SERPINE2 *polymorphisms among Finnish Caucasian construction workers**. Values of D' are shown. The haplotype structure was estimated by using the Haploview program, version 4.2.

Figure 1 shows haplotype block structure and pair-wise LD (D') values for the studied *SERPINE2 *SNPs. Three of the SNPs (rs729631, rs975278, and rs6748795) were found to be in tight linkage disequilibrium. Therefore, only one of these SNPs (rs729631) was included in the further statistical analyses, in addition to the rs840088 SNP which was in moderate linkage with the other three studied SNPs.

The rs729631 SNP showed a significant association with panlobular emphysema (*p *= 0.003) (Table 2). This association was further analysed by dividing the cases according to the existence of radiologic changes (Table 3). The radiologic signs of emphysema were considered either subnormal if the radiologic score was less than one (less than two in emphysema sum score), or pathological if the radiologic score was one or higher (two or higher in emphysema sum score).

**Table 2:** Association between *SERPINE2 *polymorphisms, emphysema findings and pulmonary function

Phenotype	SNP	β^#^	*p*-value
Emphysema score	rs729631	0.018	0.570
	rs840088	-0.016	0.614
Centrilobular	rs729631	0.000	0.987
	rs840088	0.008	0.794
Paraceptal	rs729631	-0.025	0.440
	rs840088	-0.015	0.637
Panlobular	rs729631	0.094	0.003*
	rs840088	-0.029	0.369
Bullae	rs729631	-0.010	0.767
	rs840088	-0.028	0.383
FEV_1_	rs729631	-0.028	0.377
	rs840088	-0.002	0.942
FVC	rs729631	-0.044	0.182
	rs840088	0.025	0.441
FEV_1_/FVC	rs729631	0.004	0.892
	rs840088	-0.047	0.124
DL_CO_	rs729631	-0.032	0.310
	rs840088	0.014	0.648
DL_CO_/VA	rs729631	-0.011	0.738
	rs840088	-0.026	0.401

DL_CO _= Single breath diffusing capacity for carbon monoxide, % of predicted; DL_CO/_VA = specific diffusing capacity, % of predicted; FEV_1 _= Forced expiratory volume in 1 second, % of predicted; FVC = forced vital capacity, % of predicted; ^#^Standardized coefficient β; *p < 0.05. Covariates included in analysis: Age, sex, PYs, years of asbestos exposure (emphysema and subtypes); PYs and years of asbestos exposure (FEV_1_, FVC, DL_CO_, and DL_CO_/VA); age, sex, PYs, years of asbestos exposure, height (FEV_1_/FVC).

**Table 3:** Distribution of radiologic signs in relation to emphysema subtypes

	Radiologic signs			
	
Phenotype	**No changes**^**#**^ N (%)	Any changes* N (%)	**Subnormal changes**^**¶**^ N (%)	**Pathological changes**^**+**^ N (%)
**Emphysema**^§^	599 (63.0)	352 (37.0)	231 (24.3)	121 (12.7)
**Centrilobular**	723 (76.0)	228 (24.0)	105 (11.0)	123 (12.9)
**Paraceptal**	781 (82.1)	170 (17.9)	94 (9.9)	76 (8.0)
**Panlobular**	783 (82.3)	168 (17.7)	109 (11.5)	59 (6.2)
**Bullae**	826 (86.9)	125 (13.1)	90 (9.5)	35 (3.7)

^#^Number of subjects with no emphysematous changes

*Number of subjects with emphysema score positive

^¶^Number of subjects with emphysema score less than one (less than two in emphysema sum score)

^+^Number of subjects with emphysema score one or higher (two or higher in emphysema sum score)

In the stratified analysis, the homozygous variant genotype of rs729631 SNP was found to pose over 2-fold risk for panlobular emphysema (OR 2.22, 95% CI 1.05-4.72) (Table 4). The risk was slightly increased also for the heterozygotes (OR 1.66, 95% CI 1.15-2.38), and it appeared to be mainly attributable to the pathological emphysematous changes; the ORs were 2.19 (95% CI 1.23-3.91) for heterozygous carriers of the variant allele of rs729631 SNP, and 4.37 (95% CI 1.61-11.86) for homozygous carriers of the allele. In contrast, the risk for developing subnormal panlobular emphysema was not statistically significantly associated with this SNP (Table 4).

**Table 4:** Distribution of *SERPINE2 *genotypes according to the existence and severity of panlobular emphysematous changes

Genotype	**No radiologic changes**^**#**^ N (%)	Radiologic changes* N (%)	**OR (95% CI)**^**¶**^	**Subnormal changes**^**+**^ N (%)	**OR (95% CI)**^**¶**^	**Pathologcal changes**^**++**^ N (%)	**OR (95% CI)**^**¶**^
rs729631							
G/G	500 (64.6)	85 (51.2)	1.0	60 (56.1)	1.0	25 (42.4)	1.0
G/C	245 (31.7)	70 (42.2)	1.66 (1.15-2.38)	42 (39.3)	1.43 (0.93-2.20)	28 (47.4)	2.19 (1.23-3.91)
C/C	29 (3.7)	11 (6.6)	2.22 (1.05-4.72)	5 (4.7)	1.43 (0.52-3.92)	6 (10.2)	4.37 (1.61-11.86)

^#^Number of subjects with no emphysematous changes

*Number of subjects with emphysema score positive

^¶^Adjusted for age, sex, PYs, and years of asbestos exposure

^+^Number of subjects with emphysema score less than one (less than two in emphysema sum score)

^++^Number of subjects with emphysema score one or higher (two or higher in emphysema sum score)

The significant associations were further analysed by stratifying the study population according to smoking history. No notable differences were, however, seen between mild and moderate/heavy smokers in the risk of developing emphysematous changes of panlobular type associated with certain *SERPINE2 *genotypes (data not shown).

The associations between polymorphisms and pulmonary changes were studied also separately in both original study populations (see Additional file 1). The *SERPINE2 *rs729631 SNP, which was associated with panlobular emphysema in the whole study population, showed statistical significance also in both sub-cohorts. No other significant associations were seen in the separate sub-cohorts.

In haplotype analysis of rs729631 and rs840088 SNPs, four different haplotypes were identified. The most common of these was GC (wild type-wild type, 55.0%), followed by GT (24.4%), CC (16.9%), and CT (3.7%).

In a combination analysis, the CC-haplotype (variant allele for rs729631, wild type allele for rs840088) showed almost a 1.5-fold risk for overall panlobular emphysema (OR 1.41, 95% CI 1.04-1.92) and over two-fold risk for pathological panlobular changes (OR 2.23, 95% CI 1.41-3.54) in comparison to the most common haplotype with the wild type allele for both SNPs (GC) (Table 5). The haplotype with a variant allele for both SNPs (CT) showed almost a four-fold risk for overall panlobular changes (OR 3.72, 95% CI 1.56-8.90), and subnormal panlobular changes (OR 3.98, 95% CI 1.55-10.20) in comparison to the most common haplotype (GC).

**Table 5:** Distribution of *SERPINE2 *haplotypes according to the existence and severity of panlobular emphysematous changes

**Haplotype**^**#**^	No radiologic changes* N (%)^**¶**^	**Radiologic changes**^**+**^ **N (%)**^**¶**^	**OR (95% CI) **^**++**^	**Subnormal changes**^**± **^ **N (%)**^**¶**^	**OR (95% CI) **^**++**^	**Pathological changes**^**§**^ **N (%)**^**¶**^	**OR (95% CI) **^**++**^
GC^ψ^	834 (53.3)	165 (49.1)	1.0	117 (53.7)	1.0	48 (40.7)	1.0
GT	429 (27.4)	79 (23.5)	0.92 (0.68-1.24)	49 (22.5)	0.80 (0.56-1.14)	30 (25.4)	1.20 (0.74-1.94)
CC	289 (18.5)	83 (24.7)	1.41 (1.04-1.92)	45 (20.6)	1.09 (0.75-1.59)	38 (32.2)	2.23 (1.41-3.54)
CT	14 (0.9)	9 (2.7)	3.72 (1.56-8.90)	7 (3.2)	3.98 (1.55-10.20)	2 (1.7)	2.71 (0.58-12.59)

^#^Composed of two SNPs: rs729631 and rs840088

^ψ^Wild type-wild type

*Number of subjects with no emphysematous changes

^¶^Data are presented as number of chromosomes (%)

^+^Number of subjects with emphysema score positive

^++^Adjusted for age, sex, PYs, and years of asbestos exposure

**^± ^**Number of subjects with emphysema score less than one (two or higher in emphysema sum score)

**^§^**Number of subjects with emphysema score one (two or higher in emphysema sum score)

We did not find a correlation between the studied *SERPINE2 *SNPs and lung function. However, FEV_1 _tended to be lowest in carriers of the homozygous CC variant rs729631 genotype (GG 77.8%, CG 73.7%, and CC 70.2% of predicted, respectively) and FVC was significantly lower (*p *= 0.011) in carriers of the CC genotype compared to the GG genotype (94.1%, 85.7% and 81.3% of predicted, respectively).

## Discussion

*SERPINE2 *has previously been identified as a positional candidate gene for COPD from a broad linkage region of chromosome 2q [6]. Since then, several studies have associated *SERPINE2 *polymorphisms to COPD in different populations [6-8].

We examined further the role of *SERPINE2 *polymorphisms in the development of pulmonary disorders, and report here a significant association between *SERPINE2 *SNPs and panlobular emphysema. These associations were observed in the whole study population and independently in both studied sub-cohorts. Two study groups have recently reported an association between *SERPINE2 *and emphysema [11,12], but no previous study has assessed the role of *SERPINE2 *polymorphisms in structurally different emphysema subtypes.

In the current study, the stratified analysis revealed a two-fold risk for developing panlobular emphysema for homozygous variant genotypes of *SERPINE2 *rs729631 SNP. An elevated risk was also observed for the heterozygous carriers of the variant allele of this SNP. Moreover, the risk was as high as four-fold when only pathological changes were considered. In addition, an increased risk was detected also for a haplotype composed of the variant alleles of both rs729631 and rs840088 SNPs.

All SNPs analyzed in this study have previously been associated to COPD and related phenotypes [6,7,11,12]. However, the findings vary between different studies; some studies have even reported lack of association between any of the studied *SERPINE2 *SNPs and COPD [9,10].

There are several potential explanations for the contradictory results. One important aspect is the genetic heterogeneity; different *SERPINE2 *SNPs may be involved in different populations and the pathology of the disease may also differ between the populations. Another important issue to consider in this context is the phenotypic heterogeneity. In most of the previous studies the COPD patients have been chosen based on spirometric measurements. Therefore, the cases in these study populations include subjects with and without emphysema.

In addition to the current study, two previous studies have reported association between *SERPINE2 *genotypes and emphysema [11,12]. Moreover, the study subjects in the original study by DeMeo *et al. *[6] were subsequently declared to have emphysema [22]. Consequently, the results from all these three earlier studies [6,11,12] and the present one actually converge. This suggests that the disease phenotype behind the observed association between *SERPINE2 *and COPD might indeed be structural emphysema.

The present results indicate that FEV_1 _and FVC would be lower in carriers of the *SERPINE2 *rs729631 CC genotype than in carriers of the GG genotype, whereas no association was found between the *SERPINE2 *genotypes and gas transfer capacity (DL_CO _or DL_CO_/VA), which is usually decreased in emphysema. This may partly be explained by the examination methodology: HRCT is very sensitive method and emphysematous changes can be detected before any drastic decline in lung function occurs. In addition, in AAT-deficiency, which resembles the SERPINE2-deficiency, decline in spirometric variables has been found to be an early phenomenon, whereas the decline in gas transfer capacity occurs in later stage of the disease [23]. This may well be also the case with the SERPINE2, which could explain the lack of association with gas transfer in the current study.

SERPINE2 belongs to the same serpin-superfamily of proteins as AAT, a gene deficiency of which is known to cause emphysema, especially the panlobular type emphysema. Both SERPINE2 and AAT are known to inhibit several serine proteases [24-26]. They have not, however, been demonstrated to share substrate specificity. It has been proposed that SERPINE2 could contribute to the development of COPD through interaction with matrix metalloproteinases (MMPs) [6]. However, the exact mechanistic link between panlobular changes and SERPINE2 remains to be elucidated.

Our study also has some potential limitations. First, the patients were enrolled in three cities during two separate primary studies, and therefore four different HRCT scanners had to be used and as many as seven different radiologists participated in the image reading. However, since the Finnish population is very homogenous and the three big cities, where the patients were enrolled, are all located in the southern Finland near to each other, we do not believe that geographic origin at the time of the examination has caused any significant bias in the data analysis. Moreover, any inconsistency in image reading causes inaccuracy and thus random noise to the results leading in loss of power rather than in a systematic error.

Second, our study subjects have been selected based on their asbestos exposure, which itself appeared not to be a significant predictor of emphysematous changes in the logistic regression model (data not shown). Moreover, it is highly likely that the study subjects have been occupationally exposed to other particles, such as dust, which can contribute to the development of emphysema. Unfortunately, dust exposure data was not available. However, our patient material was considerably large and a lot of ex- and current smokers were included in it. This is useful in demonstrating the genetic predisposition to emphysema, which probably would not have manifested to such degree without smoking.

Third, the multiple comparisons performed increase the possibility of detecting false positive associations. We did not perform any correction for multiple testing for a couple of reasons. First, most of the methods correcting for multiple testing are very conservative, and it is not clear, *e.g.*, what is the number of comparisons you should adjust for [27]. In addition, based on previous findings, we had an *a priori *hypothesis for each polymorphism chosen, which reduces the need for correction. Yet, these results self-evidently should be considered with caution until replicated in another study population.

The fact that only four *SERPINE2 *SNPs were examined in the current study could also be considered as a potential limitation. However, these SNPs were chosen based on several previous association studies, and two of them (rs729631 and rs975278) have been associated to COPD and related phenotypes in four different reports [6,7,11,12]. In addition, due to the limited number of women in the study the findings should naturally be generalized to females with caution.

Currently, functional consequences of none of the studied SNPs are known. It is, however, possible that the intronic areas where these SNPs reside are important regulatory elements for the transcription and translation of the gene. Alternatively, the associations observed may originate from LD with other yet unidentified susceptibility genes in the 2q area.

## Conclusions

In conclusion, our findings support the suggested association between *SERPINE2 *genotypes and development of pulmonary emphysema. As a novel finding, our study suggests that the *SERPINE2 *gene polymorphisms may be involved particularly in the development of panlobular changes.

## List of Abbreviations

AAT: alpha-1-antitrypsin; ASBE: study cohort of asbestos exposed workers recruited in 1996-1997; ASSE: study cohort of asbestos exposed workers recruited in 2003-2004; COPD: chronic obstructive pulmonary disease; DL_CO_: single breath diffusing capacity for carbon monoxide, % of predicted; DL_CO_/VA: specific diffusing capacity, % of predicted; FEV_1_: forced expiratory volume in 1 second, % of predicted; FVC: forced vital capacity, % of predicted; HRCT: high resolution computed tomography; HWE: Hardy-Weinberg equilibrium; LD: Linkage disequilibrium; MMP: matrix metalloproteinase; PY: pack-years; SERPINA1: serpin peptidase inhibitor, clade A, member 1; SERPINE2: serpin peptidase inhibitor, clade E, member 2; SNP: single nucleotide polymorphism.

## Competing interests

The authors declare that they have no competing interests.

## Authors' contributions

MKK carried the main responsibility of the genotyping, data analyses, and preparation of the manuscript; ET participated in the genotyping analyses and manuscript preparation; SH participated in the haplotype analyses and manuscript preparation; TV participated in the data collection, radiological examinations, data analysis, and manuscript preparation; PO participated in the data collection and manuscript preparation; PP participated in data collection, lung function examinations, and manuscript preparation; AH was responsible for the study design and supervision of the genotyping, data analysis, and the manuscript preparation.

All authors have read and approved the final version of the manuscript.

## Pre-publication history

The pre-publication history for this paper can be accessed here:

http://www.biomedcentral.com/1471-2350/12/157/prepub

## Supplementary Material

Additional file 1 **Table S1 - Association between *SERPINE2 *polymorphisms, emphysema findings and pulmonary function separately in both case cohorts**. Click here for file
