# Supplementary material for: SERPINE2 haplotype as a risk factor for panlobular type of emphysema
Source: BMC Med Genet. 2011 Dec 7;12:157. doi: 10.1186/1471-2350-12-157 (PMC3269992; doi:10.1186/1471-2350-12-157)
Supplement: Additional file 1 — Table S1 - Association between SERPINE2 polymorphisms, emphysema findings and pulmonary function separately in both case cohorts. [file 1471-2350-12-157-S1.DOC]

**Table S1 -** Association between *SERPINE2* polymorphisms, emphysema findings and pulmonary function separately in both case cohorts

| **Phenotype** | **SNP** | **ASBE** | | **ASSE** | |
| --- | --- | --- | --- | --- | --- |
|  |  | **β^#^** | ***p*-value** | **β^#^** | ***p*-value** |
| Emphysema score | rs729631 | 0.034 | 0.412 | -0.015 | 0.750 |
|  | rs840088 | -0.033 | 0.424 | 0.018 | 0.703 |
| Centrilobular | rs729631 | 0.035 | 0.383 | -0.052 | 0.276 |
|  | rs840088 | 0.011 | 0.786 | -0.009 | 0.859 |
| Paraceptal | rs729631 | -0.011 | 0.795 | -0.053 | 0.287 |
|  | rs840088 | -0.058 | 0.158 | 0.050 | 0.309 |
| Panlobular | rs729631 | 0.100 | 0.017^*^ | 0.101 | 0.047^*^ |
|  | rs840088 | -0.033 | 0.432 | -0.010 | 0.843 |
| Bullae | rs729631 | -0.024 | 0.566 | 0.065 | 0.212 |
|  | rs840088 | -0.045 | 0.288 | 0.049 | 0.339 |
| FEV_1_ | rs729631 | -0.059 | 0.154 | 0.023 | 0.647 |
|  | rs840088 | -0.024 | 0.553 | 0.038 | 0.439 |
| FVC | rs729631 | -0.054 | 0.202 | -0.017 | 0.748 |
|  | rs840088 | -0.007 | 0.871 | 0.088 | 0.085 |
| FEV_1_/FVC | rs729631 | -0.038 | 0.343 | 0.079 | 0.115 |
|  | rs840088 | -0.040 | 0.317 | -0.080 | 0.104 |
| DL_CO_ | rs729631 | -0.049 | 0.230 | -0.003 | 0.957 |
|  | rs840088 | -0.007 | 0.859 | 0.051 | 0.299 |
| DL_CO_/VA | rs729631 | -0.031 | 0.446 | 0.022 | 0.657 |
|  | rs840088 | -0.011 | 0.789 | -0.053 | 0.289 |

DL_CO_ = Single breath diffusing capacity for carbon monoxide, % predicted; DL_CO_ /VA = specific diffusing capacity, % predicted; FEV_1_ = Forced expiratory volume in 1 second, % predicted; FVC = forced vital capacity, % predicted

^#^Standardized coefficient β

^*^p< 0.05

Covariates included in analysis: Age, sex, pack-years, years of asbestos exposure (emphysema and subtypes); age, sex, pack-years~~,~~ and years of asbestos exposure (FEV_1_, FVC, DL_CO_, DL_CO_/VA); age, sex pack-years, years of asbestos exposure, height (FEV_1_/FVC)
